# Antibiogram at a Rural Hospital Against the Background of COVID-19: A Five-Year Retrospective Review

**DOI:** 10.7759/cureus.27221

**Published:** 2022-07-25

**Authors:** Okelue E Okobi, Endurance O Evbayekha, Hameed O Shittu, Ifeanyi E Arinze, Chukwudike G Nnaji, Nneka J Umeh, Temitope O Ajayi, Olamide O Ajayi, Oghenetega E Ayisire, Anthony I Dick, Ogochukwu Agazie, Chinelo Igweike, Chinwendum U Ekpemiro, Boma E Jacks, Nkemputaife P Onyechi

**Affiliations:** 1 Family Medicine, Lakeside Medical Center, Belle Glade, USA; 2 Internal Medicine, St. Luke's Hospital, St. Louis, USA; 3 Internal Medicine, Federal Medical Centre, Abeokuta, NGA; 4 Internal Medicine, Weiss Memorial Hospital, Chicago, USA; 5 Internal Medicine/Family Medicine, Windsor University School of Medicine, Chicago, USA; 6 General Medicine, Brooklyn Queens Nursing Home, New York, USA; 7 Internal Medicine, University at Buffalo, New York, USA; 8 Internal Medicine, Obafemi Awolowo College of Health Sciences, Olabisi Onabanjo University, Sagamu, NGA; 9 Psychiatry, University of South Wales, Pontypridd, GBR; 10 Public Health, Chicago State University, Chicago, USA; 11 General Medicine, College of Medicine, University of Lagos, Idi Araba, NGA; 12 Preventive Medicine, RBH Medical Center, Richmond, USA; 13 Surgery, Federal Medical Centre, Umuahia, NGA; 14 Surgery, University of Maiduguri Teaching Hospital, Maiduguri, NGA; 15 Internal Medicine, University Hospitals Cleveland Medical Center, Cleveland, USA

**Keywords:** antibiogram, antimicrobial agent, bacterial susceptibility, pre-covid-19, post-covid-19

## Abstract

Background and objective

The role of the antibiogram in reducing hospital length of stay (LOS), mortality rate, health care costs, and, by extension, patients' social, physical, and emotional wellness has a significant impact on the medical community. Hospitals in large cities serve a dynamic population of diverse ethnic groups. Many scholarly works and publications have shown that the antimicrobial pattern in rural settings has significant variability annually. Over the last two years, the spread of coronavirus disease 2019 (COVID-19) has brought about many unknowns in the sphere of healthcare. The pattern of pathology accompanying COVID-19 has affected hospital policies and direct patient management, leading to a paradigm shift in approaches, policies, and resource utilization. The years 2019 to 2021 were marked by many admissions due to COVID-19, and the effects of COVID-19 are still being studied. In light of this, this study examined the changes in sensitivity patterns, new trends, and nature of bacteria isolates, antimicrobial rates, and susceptibility based on a rural hospital’s annual antibiogram pertaining to its central departments: the intensive care unit (ICU), patient care unit (PCU), the outpatient unit, and emergency department (ED).

Methods

This five-year retrospective antibiogram review compared antibiogram patterns two years before the first case of COVID-19 was reported in the hospital and those two years after the initial outbreak.

Results

The organism comparative susceptibility tests for *Escherichia coli (E. coli)* were not significant except for increased susceptibility toward nitrofurantoin (p=0.003); *Klebsiella pneumoniae* (*K. pneumoniae)* was also not significant except for the increased susceptibility to ciprofloxacin (p=0.003). *Pseudomonas aeruginosa (P. aeruginosa)* had no changes in susceptibility patterns, while *Proteus mirabilis (P. mirabilis) *had increased susceptibility to imipenem (p=0.05), aztreonam (p=0.00), and meropenem (p=0.004), with reduced susceptibility to gentamicin (97.47% vs. 88.24%, p=0.006). There was a whopping decrease in the sensitivity of methicillin-resistant *Staphylococcus aureus* (MRSA) to clindamycin (75.93% vs. 50.7%, p=0.000), linezolid (99.54% vs. 88.73, p=0.004), trimethoprim/sulfamethoxazole (92.59% vs. 74.65%, p=0.001), and vancomycin (99.54% vs. 88.73%, p=0.004). *Staphylococcus aureus (S. aureus)* had no significant variation except an increase in susceptibility to nitrofurantoin (p=0.023), and perhaps ironically, *Streptococcus pneumoniae (S. pneumoniae)* had no significant changes in susceptibility pattern.

Conclusion

Our data demonstrate that the susceptibility of different drugs against different bacterial pathogens varied. However, some antibiotic drugs were found to have high susceptibility against different isolated organisms, and these drugs include amikacin, levofloxacin, vancomycin, cefotaxime, nitrofurantoin, and ceftriaxone. Some organisms showed a significantly declined antibiotic susceptibility, while others showed a significant improvement. The role of COVID-19 regarding these changes is unknown. COVID-19 may not be the cause of the observed differences. We believe that further research on antibiotic legislation and prescribing trends is required. Other non-significant study findings may be attributed to the limited data available to us.

## Introduction

The antibiogram represents the summary of bacterial pathogen susceptibility to different antimicrobial agents, and it is usually generated in a tabular form. This vital hospital record documents microbial susceptibility and antibiotic resistance trends in specific healthcare settings. The antibiogram is an essential resource for institutions to track changes in antimicrobial resistance (AMR) and guide empirical antimicrobial therapy [1]. Clinicians use antibiograms to assess local susceptibility rates; they also aid in selecting empiric antibiotic therapy and monitoring resistance trends over time within an institution/facility. Antibiograms are generated from bacterial isolates (from patients' tissues or body fluids) and subjected to laboratory testing [1-2]. These data are collated periodically from culture and sensitivity studies done on samples taken from patients treated for microbial infections in a hospital. Antibiograms could also be helpful during epidemics when there is high usage of antibiotics or other drugs.

Annual antibiograms are hospital datasets showcasing bacterial isolates and their antibiotic susceptibility pattern for a particular year. These results are often publicly presented annually to clinicians within the hospital [1]. The data are then analyzed to create policies that guide best practices in selecting antimicrobials, analyzing susceptibility patterns, determining new trends, and formulating policies needed for the hospital's administrative and accreditation purposes. Bacterial cultures and resistance patterns assess mortality and morbidity rates in a hospital reasonably well. The role of antibiograms in reducing hospital stay, mortality rates, healthcare costs, and by extension, patients' social, physical, and emotional wellness cannot be emphasized enough [1]. Our hospital serves a rural health workforce from a diverse ethnic group. Many scholarly articles have demonstrated that disease patterns in rural settings vary significantly from their urban counterparts [3-4].However, from 2019 to 2021, the spread of coronavirus disease 2019 (COVID-19) has ushered in many unknowns in the field of healthcare. The patterns of pathology accompanying COVID-19 have affected hospital policies and direct patient management and led to a paradigm shift in approaches, policies, and resource utilization.

We hypothesize that certain microbial and susceptibility patterns are predictable with a few outliers. The likelihood of variability in the outcome of an antibiogram can be confounded by various factors, including the nature of the practice, patients' socioeconomic background and prevailing diseases, and hospital sepsis policy. The effect of COVID-19 on antibiograms still remains an enigma, and we believe the findings of this analysis will contribute to the design and implementation of best practices and policies.

## Materials and methods

The objective of the study was to compare the patterns related to rates of antimicrobials and their susceptibility in our hospital's central departments: the intensive care unit (ICU), patient care unit (PCU), the outpatient unit, and emergency department (ED). The antibiogram from this rural primary care center was studied and analyzed to see if there are new patterns emerging against the background of the COVID-19 outbreak in the past few years.

We hypothesize that isolates will exhibit different antimicrobial susceptibilities in the context of COVID-19 compared to susceptibility and sensitivity patterns that were documented two years before the COVID-19 pandemic, as reported by the previous hospital-wide antibiogram.

This study involved a five-year retrospective antibiogram review of our rural primary care center during the COVID-19 pandemic (2019-2021). Our inclusion and exclusion criteria were as described in Table 1 below. The dataset was compared to that in the two years prior to the outbreak of the pandemic (2017-2019) (Table 2). The data were compiled, checked, and analyzed using SPSS Statistics version 26 (IBM Corp., Armonk, NY); we used Fisher's exact test and Chi-square test to analyze the data collected from the antibiogram to determine differences and significances. Details of the empiric treatment with antibiotics were also recorded as secondary data.

**Table 1:** Selection criteria for the study

Inclusion criteria	Exclusion criteria
Antibiograms from 2017 to 2021 at our rural healthcare center	Repeat cultures
	Antibiograms before 2017 or after 2021

**Table 2:** Percentage (%) susceptibility trends over the years *E. coli: Escherichia coli; K. pneumoniae: Klebsiella pneumoniae; P. mirabilis: Proteus mirabilis; E. cloacae: Enterobacter cloacae; P. aeruginosa: Pseudomonas aeruginosa; E. faecalis: Enterococcus faecalis; S. aureus: Staphylococcal aureus;* CoNS: coagulase-negative staphylococci; *S. pneumoniae: Streptococcus pneumoniae*

Years	E. coli	K. pneumoniae	P. mirabilis	E. cloacae	P. aeruginosa	E. faecalis	S. aureus	CoNS	S. pneumoniae	
2017	484	93	70	71	57	71	55	36	6	
2018	494	120	61	80	41	39	60	39	6	
2019	535	124	67	65	26	51	43	21	5	
2020	490	119	54	53	44	30	50	28	9	
2021	409	112	48	19	44	57	33	52	12	
Total	2412	568	300	288	212	248	241	176	38	4483

In this retrospective study conducted at a rural hospital in Florida, we analyzed 4483 positive cultures, during two contrasting periods of two years each, before and during the COVID-19 pandemic. The most common pathogen observed was *Escherichia coli (E. coli) *(2412, 53.8%), followed by *Klebsiella* spp. (568, 12.67%). *Enterococcus* spp. (i.e., *cloaca and faecalis*) was the leading Gram-positive pathogen (285, 11.95%).

## Results

The results from the antibiogram analysis helped in determining the percentages, susceptibility patterns, and significant changes compared to previously existing data within the hospital before the outbreak of COVID-19. 

**Figure 1:**
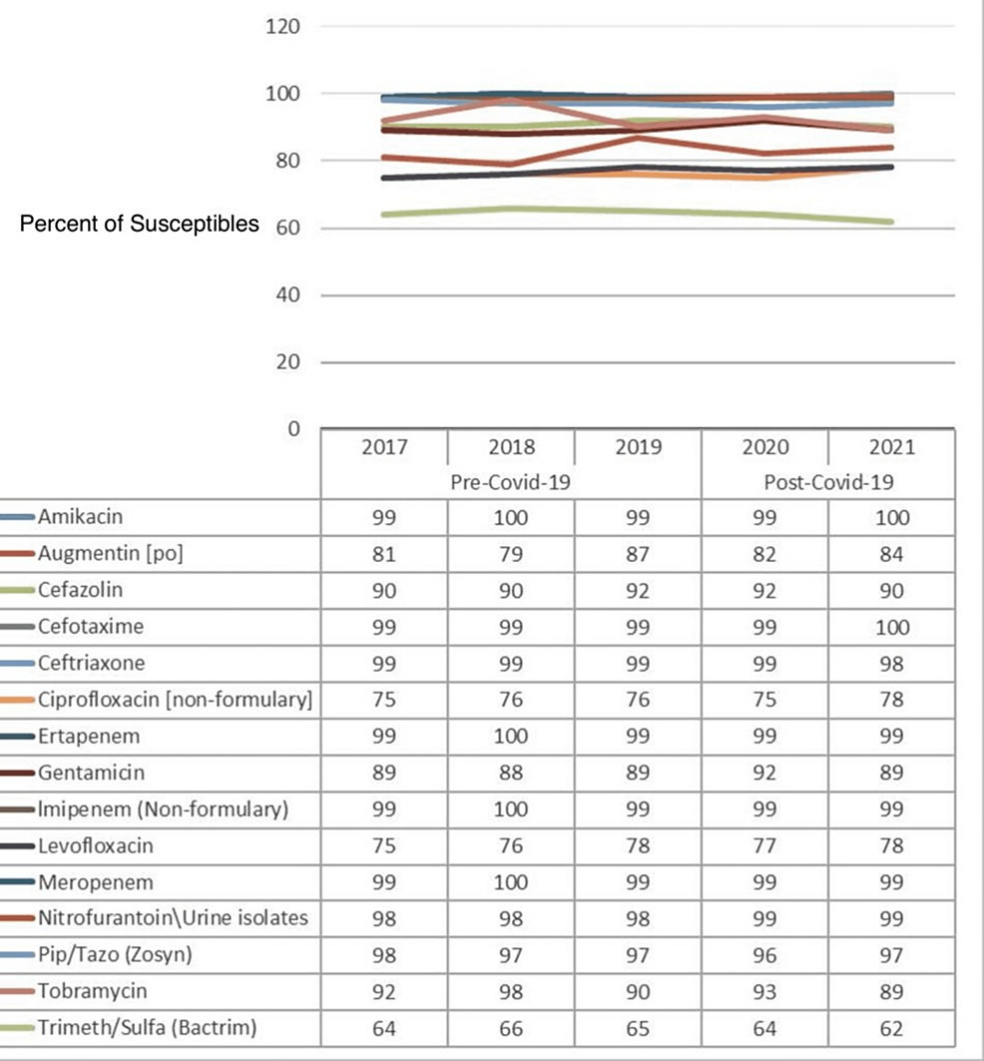
Pre- and post-COVID-19 trend comparison of the percentage of isolates susceptible to common drugs against Escherichia coli

In Figure 1, the percentage of susceptibility of common antibiotics drugs against *E. coli* is presented, and a comparison is made between the pre-COVID-19 (2017-2019) and post-COVID-19 (2020-2021) data. The data from yearly antibiograms were analyzed during these periods, and it was found that the overall susceptibility toward amikacin, cefotaxime, ciprofloxacin, gentamicin, levofloxacin, and nitrofurantoin had improved. At the same time, susceptibility to ceftriaxone, piperacillin/tazobactam (Zosyn), and tobramycin mildly decreased. While Augmentin remained relatively stable, the most effective antibiotics observed were amikacin and cefotaxime for both the pre-COVID-19 and post-COVID-19 periods for this bacteria.

As shown in Table 3, there is no evidence to prove the significance of the effect of any antibiotics against *E. coli* for the post-COVID-19 period because the p-values for all antibiotics were greater than 0.05, except for nitrofurantoin, which had a p-value of 0.033. This statistical observation could be attributed to insufficient data for this bacteria isolate.

**Table 3:** The significance of the differences in susceptibility of different antibiotic drugs against Escherichia coli in the pre-COVID-19 vs. post-COVID-19 periods *Significant as p-value <0.05; NS: non-significant as p-value >0.05

Drugs	Susceptibility against *Escherichia coli*		P-value
	Pre-COVID-19	Post-COVID-19	
				Amikacin	99.34	99.44	0.746 (NS)
Augmentin (PO)	82.42	82.98	0.724 (NS)
Cefazolin	90.75	91.1	0.769 (NS)
Cefotaxime	99.01	99.44	0.221 (NS)
Ceftriaxone	99.01	98.55	0.336 (NS)
Ciprofloxacin (non-formulary)	75.68	76.42	0.68 (NS)
Ertapenem	99.34	99	0.385 (NS)
Gentamicin	88.7	90.66	0.122 (NS)
lmipenem (non-formulary)	99.34	99	0.385 (NS)
Levofloxacin	76.34	77.42	0.122 (NS)
Meropenem	99.34	99	0.385 (NS)
Nitrofurantoin, urine isolates only (PO)	97.95	99	0.033*
Pip/tazo (Zosyn)	97.29	96.44	0.255 (NS)
Tobramycin	93.26	91.21	0.074 (NS)
Trimeth/sulfa (Bactrim)	65.04	63.18	0.359 (NS)

**Figure 2:**
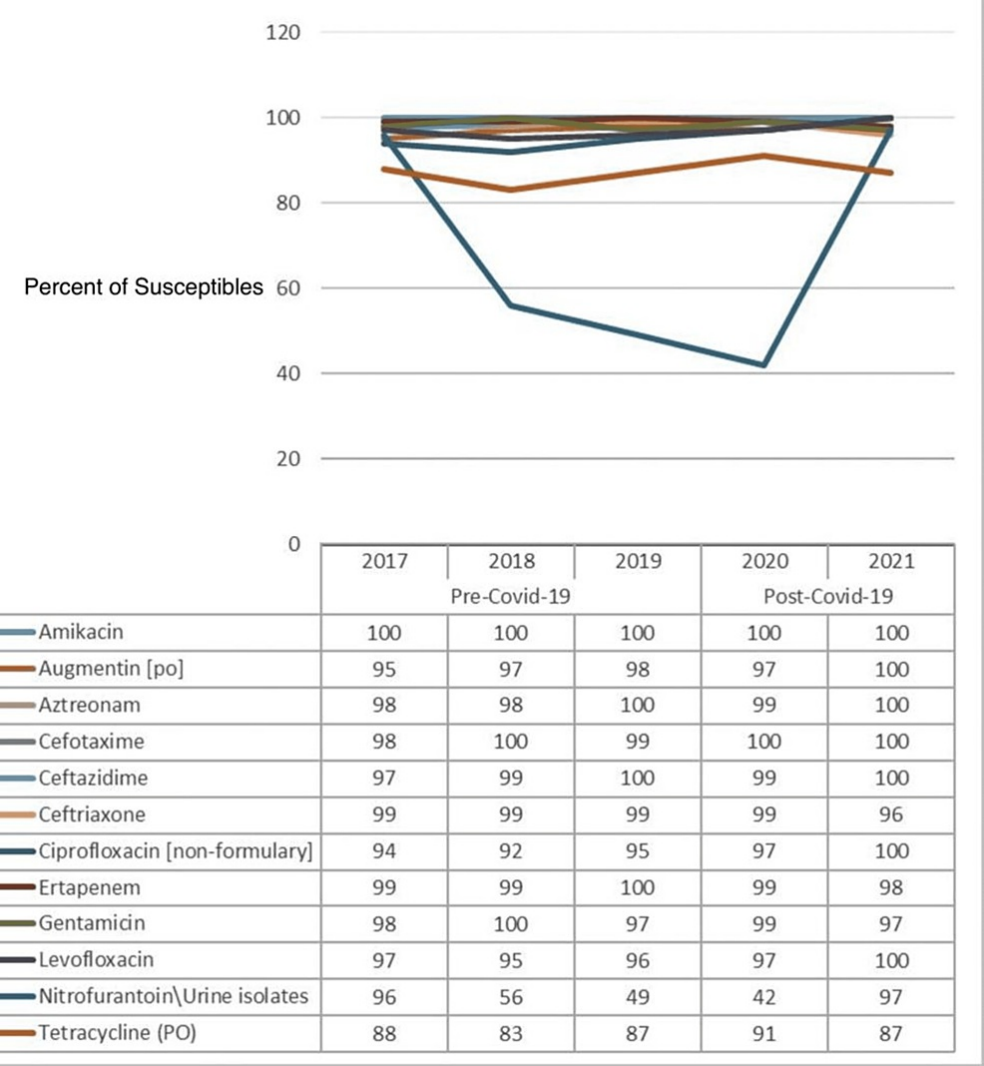
Pre- and post-COVID-19 trend comparison of the percentage of isolates susceptible to common drugs against Klebsiella pneumoniae

Figure 2 shows the percentage of susceptibility of common antibiotics drugs against *Klebsiella pneumoniae (K. pneumoniae)*, and a comparison is made between the pre-COVID-19 (2017-2019) and post-COVID-19 (2020-2021) data. The data from yearly antibiograms were analyzed during these periods and it was found that while the overall susceptibility to amikacin had remained unchanged throughout the five years, susceptibility to Augmentin, aztreonam, cefotaxime, ceftazidime, ciprofloxacin, levofloxacin, and tetracycline had increased, while susceptibility to ceftriaxone and ertapenem had decreased. The most effective antibiotics were amikacin, aztreonam, and cefotaxime for both pre-COVID-19 and post-COVID-19 periods.

As displayed in Table 4, after controlling for COVID-19, the effects of none of the antibiotics against *K. pneumoniae* can be considered statistically significant because their p-values are greater than 0.05, except for ciprofloxacin, which had a p-value of 0.003 (a p-value less than or equal to 0.05 is considered statistically significant, while that greater than 0.05 is considered not statistically significant). This statistical conclusion may be due to a shortage of data about this particular strain of bacteria.

**Table 4:** The significance of the differences in susceptibility of different antibiotic drugs against Klebsiella pneumoniae in the pre-COVID-19 vs. post-COVID-19 periods *Highly significant as p-value <0.01. NS: not significant as p-value >0.05

Drugs	Susceptibility against *Klebsiella pneumoniae*		P-value
	Pre-COVID-19	Post-COVID-19	
				Amikacin	100	100	1.000 (NS)
Augmentin (PO)	96.74	98.27	0.236 (NS)
Aztreonam	98.81	99.57	0.302 (NS)
Cefotaxime	99.11	100	0.082 (NS)
Ceftazidime	98.81	99.57	0.302 (NS)
Ceftriaxone	99.11	97.84	0.240 (NS)
Ciprofloxacin (non-formulary)	93.47	98.27	0.003^*^
Ertapenem	99.41	98.7	0.409 (NS)
Gentamicin	98.22	98.27	0.965 (NS)
Levofloxacin	95.85	98.27	0.080 (NS)
Nitrofurantoin, urine isolates only (PO)	64.39	68.83	0.268 (NS)
Tetracycline (PO)	86.05	88.74	0.338 (NS)

**Figure 3:**
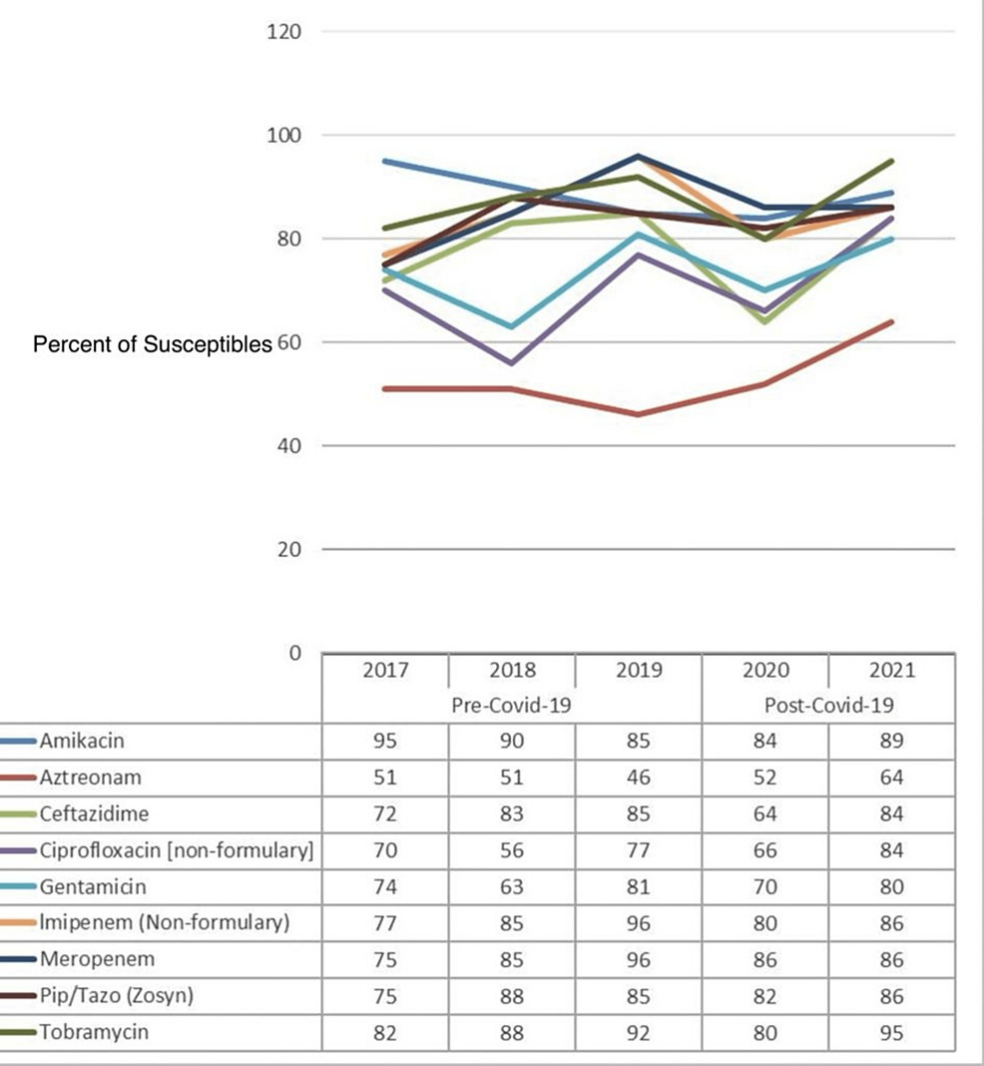
Pre- and post-COVID-19 trend comparison of the percentage of isolates susceptible to common drugs against Pseudomonas aeruginosa

Figure 3 depicts the percentage of susceptibility of common antibiotics against *Pseudomonas aeruginosa (P. aeruginosa)*, and a comparison is made between the pre-COVID-19 (2017-2019) and post-COVID-19 (2020-2021) data. The data from yearly antibiograms were analyzed during these periods, and it was found that the overall susceptibility to aztreonam, ciprofloxacin, and tobramycin had increased. While the susceptibility to gentamicin and meropenem had also increased, that to ceftazidime, imipenem, and piperacillin/tazobactam had decreased. Amikacin was the most effective antibiotic in both the pre-and post-COVID-19 phases.

As presented in Table 5, after controlling for COVID-19, the effects of none of the antibiotics against *P. aeruginosa* can be considered statistically significant because all of their p-values are greater than 0.05. This statistical conclusion may be attributed to inadequate data about this particular strain of bacteria.

**Table 5:** The significance of the differences in susceptibility of different antibiotic drugs against Pseudomonas aeruginosa in the pre-COVID-19 vs. post-COVID-19 periods NS: not significant as p-value >0.05

Drugs	Susceptibility against Pseudomonas aeruginosa		P-value
	Pre-COVID-19	Post-COVID-19	
				Amikacin	91.13	86.36	0.285 (NS)
Aztreonam	50	57.95	0.250 (NS)
Ceftazidime	78.23	73.86	0.465 (NS)
Ciprofloxacin (non-formulary)	66.94	75	0.197 (NS)
Gentamicin	71.77	75	0.599 (NS)
Imipenem (non-formulary)	83.87	82.95	0.860 (NS)
Meropenem	83.06	86.36	0.507 (NS)
Pip/tazo (Zosyn)	81.45	84.09	0.614 (NS)
Tobramycin	86.29	87.5	0.796 (NS)

**Figure 4:**
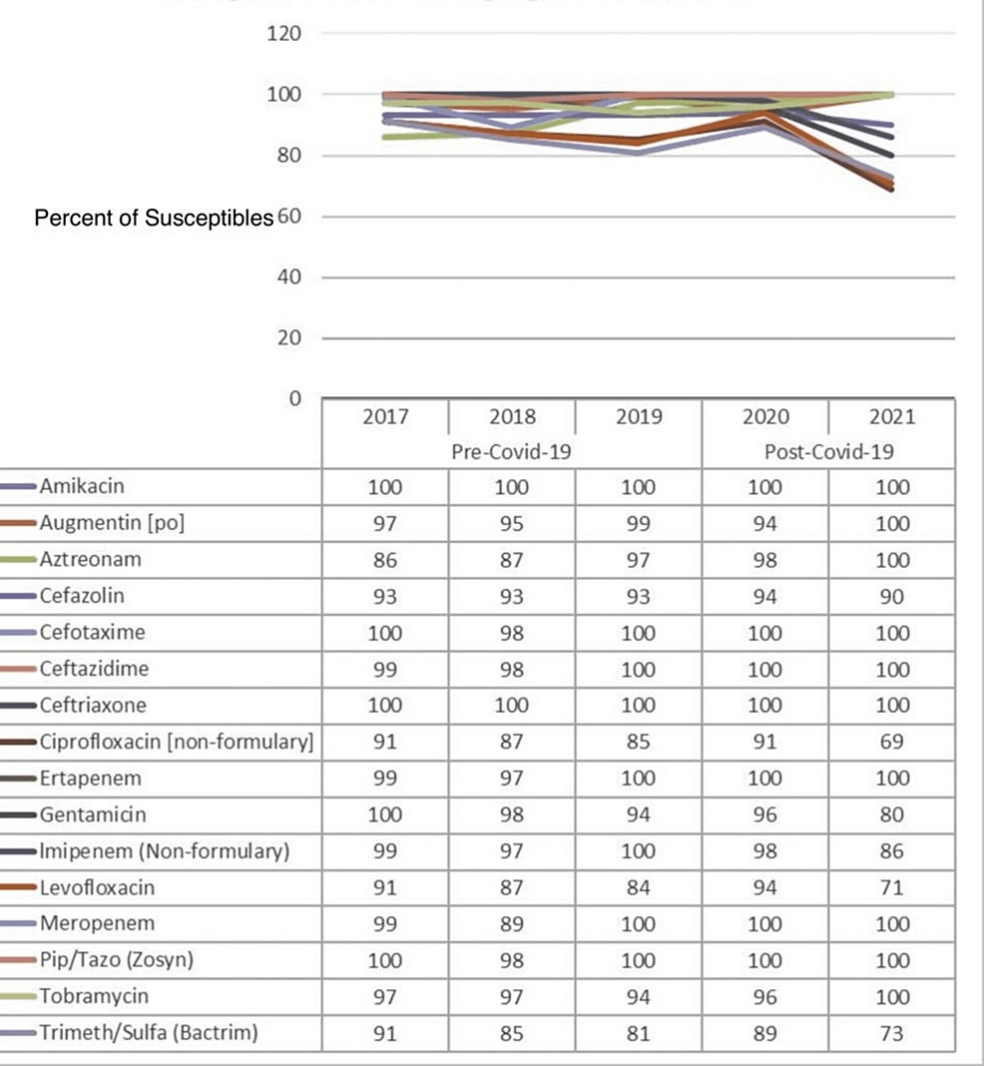
Pre- and post-COVID-19 trend comparison of the percentage of isolates susceptible to common drugs against Proteus mirabilis

Figure 4 illustrates the percentage of susceptibility of common antibiotics against *Proteus mirabilis (P. mirabilis)*, and a comparison is made between the pre-COVID-19 (2017-2019) and COVID-19 (2020-2021) periods. The data from yearly antibiograms were analyzed during these periods, and it was found that the overall susceptibility toward amikacin had remained unchanged, while that to Augmentin, aztreonam, cefotaxime, ceftazidime, ceftriaxone, ertapenem, meropenem, piperacillin, and tobramycin had increased. The susceptibility to cefazolin, levofloxacin, ciprofloxacin, gentamicin, imipenem, and trimethoprim had decreased. Amikacin was the antibiotic with the highest observed effectiveness during both the pre- and post-COVID-19 phases.

As shown in Table 6, the effects of the following antibiotics were found to be significant against *P. mirabilis:* imipenem was substantially significant with a p-value of <0.05, while aztreonam, gentamicin, and meropenem were highly significant with a p-value of <0.01 (a p-value less than or equal to 0.05 is considered statistically significant, while that greater than 0.05 is considered not statistically significant).

**Table 6:** The significance of the differences in susceptibility of different antibiotic drugs against Proteus mirabilis in the pre-COVID-19 vs. post-COVID-19 periods *Highly significant as p-value <0.01; **significant as p-value <0.05; NS: not significant as p-value >0.05

Drugs	Susceptibility against *Proteus mirabilis*		P-value
	Pre-COVID-19	Post-COVID-19	
				Amikacin	100	100	1.000 (NS)
Augmentin (PO)	96.97	97.06	0.966 (NS)
Aztreonam	89.9	99.02	0.000^*^
Cefazolin	92.93	92.16	0.811 (NS)
Cefotaxime	99.49	100	0.316 (NS)
Ceftazidime	98.99	100	0.155 (NS)
Ceftriaxone	100	100	1.000 (NS)
Ciprofloxacin (non-formulary)	87.88	80.39	0.101 (NS)
Ertapenem	98.48	100	0.081 (NS)
Gentamicin	97.47	88.24	0.006^*^
lmipenem (non-formulary)	98.48	92.16	0.024^**^
Levofloxacin	87.37	83.33	0.356 (NS)
Meropenem	95.96	100	0.004^*^
Pip/tazo (Zosyn)	99.49	100	0.316 (NS)
Tobramycin	95.96	98.04	0.289 (NS)
Trimeth/sulfa (Bactrim)	85.86	81.37	0.328 (NS)

**Figure 5:**
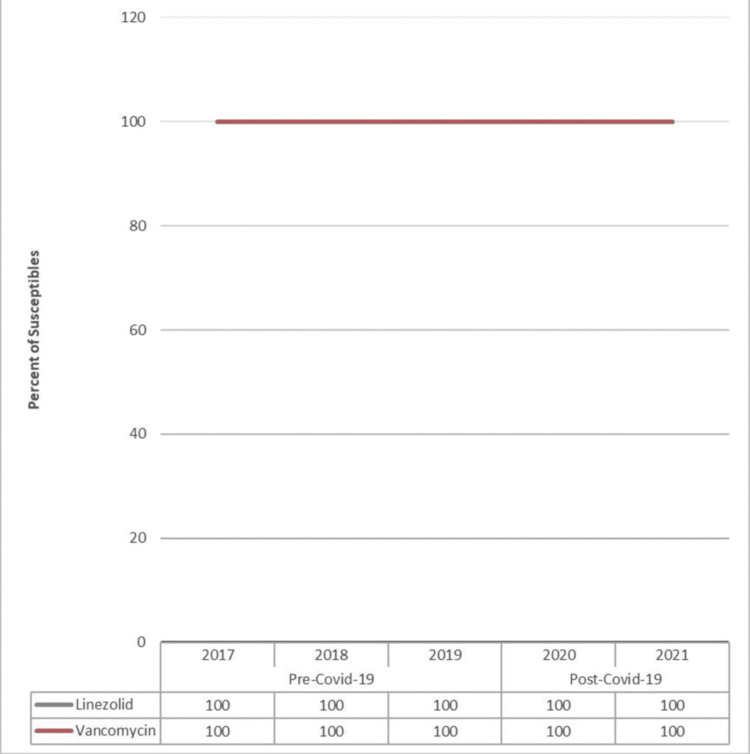
Pre- and post-COVID-19 trend comparison of the percentage of isolates susceptible to common drugs against Enterococcus faecalis

Figure 5 demonstrates the percentage of susceptibility of common antibiotic drugs against *Enterococcus faecalis (E. faecalis)*, and a comparison is made between the pre-COVID-19 (2017-2019) and post-COVID-19 (2020-2021) periods. The data from yearly antibiograms were analyzed during these periods, and it was found that the overall susceptibility to linezolid and vancomycin was stable for both periods.

As shown in Table 7, there is no statistical evidence to establish the significance of the effect of any antibiotic drug against *E. faecalis* in the post-COVID-19 period, as the p-values for all the drugs are >0.05.

**Table 7:** The significance of the differences in susceptibility of different antibiotic drugs against Enterococcus faecalis in the pre-COVID-19 vs. post-COVID-19 periods NS: not significant as p-value >0.05

Drugs	Susceptibility against *Enterococcus faecalis*		P-value
	Pre-COVID-19	Post-COVID-19	
				Linezolid	100	100	1.000 (NS)
Vancomycin	100	100	1.000 (NS)

**Figure 6:**
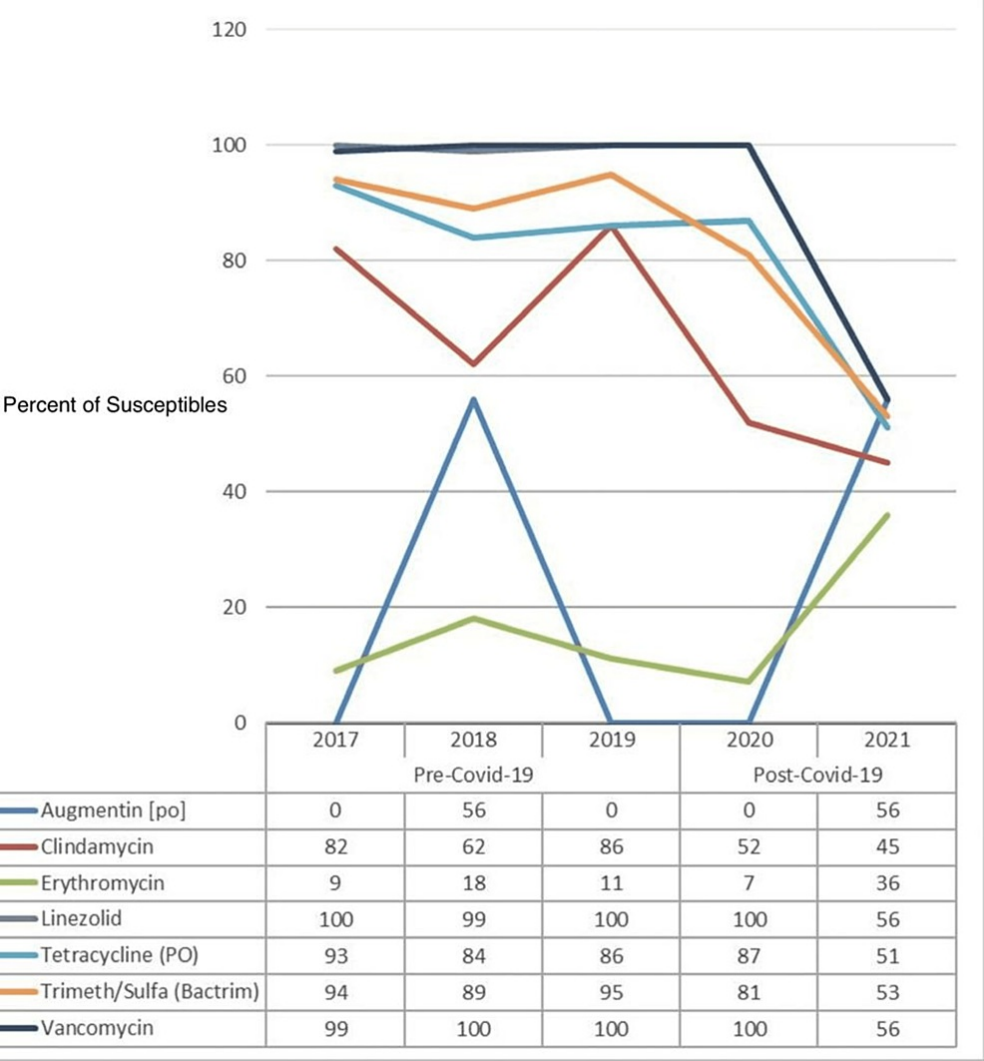
Pre- and post-COVID-19 trend comparison of the percentage of isolates susceptible to common drugs against MRSA MRSA: methicillin-resistant *Staphylococcus aureus*

Figure 6 shows the percentage of susceptibility of common antibiotics against methicillin-resistant *Staphylococcus aureus* (MRSA), and a comparison is made between the pre-COVID-19 (2017-2019) and post-COVID-19 (2020-2021) periods. The data from yearly antibiograms were analyzed during these periods, and it was found that the overall susceptibility of linezolid, tetracycline, trimethoprim, and vancomycin had decreased.

As shown in Table 8, there is no evidence to establish the significance of the effects of any antibiotic drug against MRSA for the post-COVID-19 period as the p-values for all the drugs are >0.05, except for clindamycin, linezolid, trimethoprim/sulfamethoxazole, and vancomycin, all with p-values <0.05.

**Table 8:** The significance of the differences in susceptibility of different antibiotic drugs against MRSA in the pre-COVID-19 vs. post-COVID-19 periods *Highly significant as p-value <0.01; NS: not significant as p-value >0.05 MRSA: methicillin-resistant *Staphylococcus aureus*

Drugs	Susceptibility against MRSA		P-value
	Pre-COVID-19	Post-COVID-19	
				Augmentin (PO)	20.83	14.08	0.174 (NS)
Clindamycin	75.93	50.7	0.000^*^
Erythromycin	12.5	14.08	0.736 (NS)
Linezolid	99.54	88.73	0.004^*^
Tetracycline (PO)	87.5	77.46	0.065 (NS)
Trimeth/sulfa (Bactrim)	92.59	74.65	0.001^*^
Vancomycin	99.54	88.73	0.004^*^

**Figure 7:**
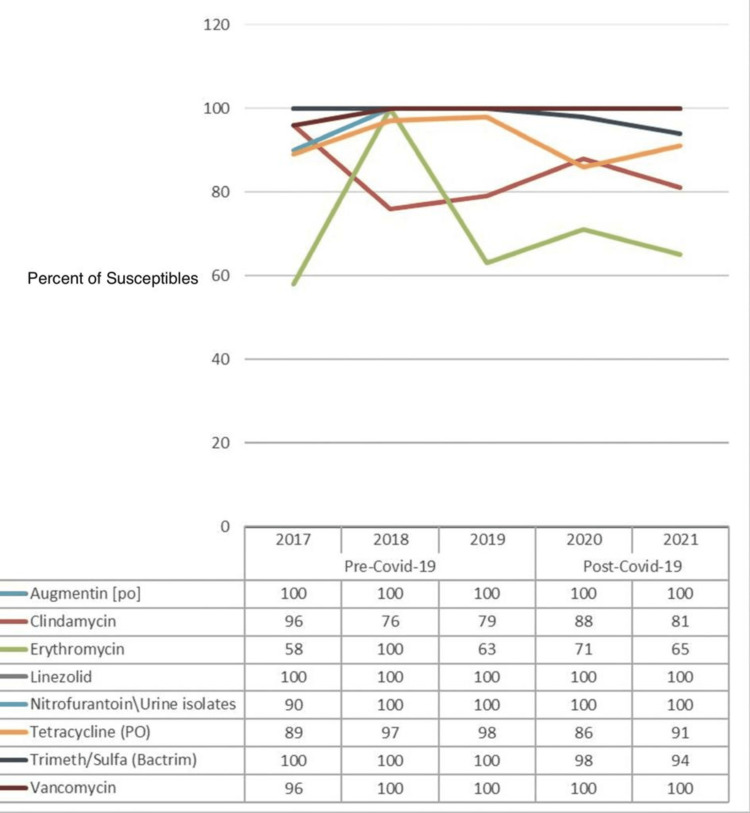
Pre- and post-COVID-19 trend comparison of the percentage of isolates susceptible to common drugs against Staphylococcus aureus

Figure 7 displays the percentage of susceptibility of common antibiotic drugs against *Staphylococcus aureus (S. aureus)*, and a comparison is made between the pre-COVID-19 (2017-2019) and post-COVID-19 (2020-2021) periods. The data from yearly antibiograms were analyzed during these periods, and it was found that the overall susceptibility to clindamycin, linezolid, nitrofurantoin, and vancomycin had increased. Linezolid had remained stable while susceptibility to erythromycin, tetracycline, and trimethoprim had decreased. The most effective antibiotics were amikacin, linezolid, nitrofurantoin, and vancomycin for both the pre-COVID-19 and post-COVID-19 phases.

As displayed in Table 9, there is no evidence to establish the significance of the effect of any antibiotic drug against *S. aureus* for the post-COVID-19 period as the p-values for all the drugs are >0.05, except for nitrofurantoin, with a p-value of 0.003. This statistical conclusion may be due to a shortage of data about this particular strain of bacteria.

**Table 9:** The significance of the differences in susceptibility of different antibiotic drugs against Staphylococcus aureus in the pre-COVID-19 vs. post-COVID-19 periods *Significant as p-value <0.05; NS: not significant as p-value >0.05

Drugs	Susceptibility against *Staphylococcal aureus*		P-value
	Pre-COVID-19	Post-COVID-19	
				Augmentin (PO)	100	100	1.000 (NS)
Clindamycin	84.18	85.54	0.777 (NS)
Erythromycin	75.32	68.67	0.279 (NS)
Linezolid	100	100	1.000 (NS)
Nitrofurantoin, urine isolates only (PO)	96.84	100	0.023^*^
Tetracycline (PO)	94.3	87.95	0.114 (NS)
Trimeth/sulfa (Bactrim)	100	96.39	0.078 (NS)
Vancomycin	98.73	100	0.155 (NS)

**Figure 8:**
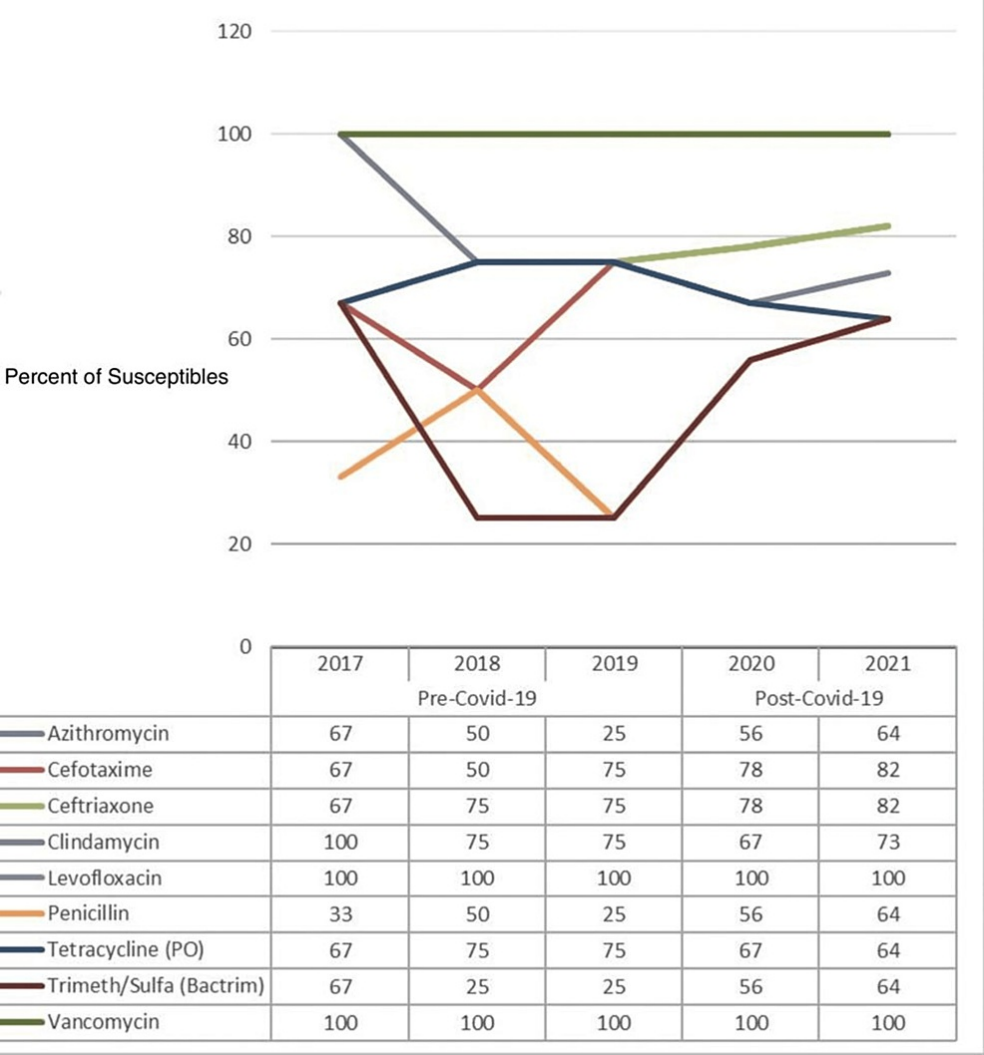
Pre- and post-COVID-19 trend comparison of the percentage of isolates susceptible to common drugs against Streptococcus pneumoniae

Figure 8 outlines the percentage of susceptibility of common antibiotic drugs against *Streptococcus pneumoniae (S. pneumoniae)*, and a comparison is made between the pre-COVID-19 (2017-2019) and post-COVID-19 (2020-2021) periods. The data from yearly antibiograms were analyzed during these periods, and it was found that the overall susceptibility to ceftriaxone and vancomycin had remained stable over the five years. In comparison, susceptibility to clindamycin and tetracycline had decreased. Before and after the spread of COVID-19, levofloxacin and vancomycin were the most effective antibiotics against this organism.

As shown in Table 10, no evidence was found to establish the significance of the effect of any antibiotic drug against *S. pneumoniae* for the post-COVID-19 period, as the p-values for all the drugs are >0.05.

**Table 10:** The significance of the differences in susceptibility of different antibiotic drugs against Streptococcus pneumoniae in the pre-COVID-19 vs. post-COVID-19 periods NS: not significant as p-value >0.05

Drugs	Susceptibility against *Streptococcus pneumoniae*		P-value
	Pre-COVID-19	Post-COVID-19	
				Azithromycin	47.06	61.9	0.356 (NS)
Cefotaxime	64.71	80.95	0.260 (NS)
Ceftriaxone	76.47	80.95	0.738 (NS)
Clindamycin	88.24	71.43	0.182 (NS)
Levofloxacin	100	100	1.000 (NS)
Penicillin	35.29	61.9	0.090 (NS)
Tetracycline (PO)	76.47	66.67	0.500 (NS)
Trimeth/sulfa (Bactrim)	41.18	61.9	0.194 (NS)
Vancomycin	100	100	1.000 (NS)

## Discussion

During the initial outbreak of the COVID-19 pandemic, a large number of individuals and patients were treated with antimicrobials, despite the outbreak being caused by a virus [4]. And although less than 12% of patients presenting with COVID-19 at the time required antibiotics, most of these patients received them nonetheless [4,5]. The spread of false information via the media contributed immensely to aggravating the scenario, raising the consumption of non-recommended antibiotics during the pandemic, resulting in close to 67% of individuals self-medicating with antimicrobials prior to their hospital presentation with viral infections [6].

The relatively high consumption of antimicrobials during the pandemic indicates that antibiotic and multidrug resistance will likely skyrocket during and after the pandemic. This directly impacts the patients and worsens morbidity and mortality among them [7,8]. Some studies have suggested that if this trend goes unchecked, about 10 million deaths are expected to occur by 2050 [9]. In light of this, our cross-sectional study based on data derived from 4483 isolates of organisms in the antibiogram examined pre- and post-COVID-19 data in order to provide an insight into the changes that have taken place over the course of the last five years.

Our study’s finding of the overall decrease in sensitivity to MRSA aligns with a study performed during the influenza A virus pandemic, which revealed a decrease in the sensitivity to cephalosporins and levofloxacin due to their excessive application during the influenza A pandemic [7]. However, in contrast to the findings of Gasperini et al., our study did not reveal a significant decrease in the susceptibility to levofloxacin. Our study’s comparative susceptibility tests for *E. Coli* were insignificant except for increased susceptibility to nitrofurantoin (p=0.003). The effect on *K. pneumoniae* was also not significant except for increased susceptibility to ciprofloxacin (p=0.003); *P. aeruginosa* had no changes in susceptibility pattern, while *P. mirabilis* had increased susceptibility to imipenem (p=0.05), aztreonam (p=0.00), and meropenem (p=0.004), with reduced susceptibility to gentamicin (97.47% vs. 88.24%, p=0.006).

We believe we should focus more on the whopping decrease in sensitivity of MRSA to clindamycin (75.93% vs. 50.7%, p=0.000), linezolid (99.54% vs. 88.73, p=0.004), trimethoprim/sulfamethoxazole (92.59% vs. 74.65%, p=0.001), and vancomycin (99.54% vs. 88.73%, p=0.004). Our study’s finding of the overall decrease in sensitivity to ceftriaxone is similar to a study done during the Influenza A virus pandemic, which revealed a decrease in the sensitivity to cephalosporins and levofloxacin as a result of excessive application of these antibiotics during the said pandemic [7]. However, in contrast to Gasperini et al., our study did not show a significant decrease in levofloxacin susceptibility.

The data from yearly antibiograms were analyzed during these periods and it was found that the overall susceptibility of amikacin, cefotaxime, ciprofloxacin, levofloxacin, and nitrofurantoin had improved. Yearly antibiograms are hospital datasets showcasing bacterial isolates and their antibiotic susceptibility patterns. These results are often publicly presented annually to clinicians within the hospital. The data is then analyzed to create policies that guide best practices in selecting antimicrobials, analyzing susceptibility patterns, determining new trends, and formulating policies for hospital administration, accreditation, and other purposes [2]. Bacterial cultures and resistance patterns determine, to a reasonable extent, mortality and morbidity rates in a hospital.

AMR, one of the major elements that antibiograms document, poses a severe global threat of growing concern to humans, animals, and environmental health. AMR occurs when microorganisms, including bacteria, viruses, fungi, and parasites, gain the ability to adapt and grow in the presence of medications that once impacted them [2]. In this study, some of the most commonly isolated organisms include *E. coli,* *K. pneumoniae*, and *P. aeruginosa*. These microbes have different resistance mechanisms to the various antimicrobials used to treat their infections.

The most problematic mechanisms in *E. coli* correspond to the acquisition of genes coding for extended-spectrum β-lactamases (conferring resistance to broad-spectrum cephalosporins), carbapenemases (conferring resistance to carbapenems), 16S rRNA methylases (conferring pan-resistance to aminoglycosides), plasmid-mediated quinolone resistance (PMQR) genes (conferring resistance to fluoroquinolones), and mcr genes (conferring resistance to polymyxins) [10].

*K. pneumoniae* is a common cause of multidrug‐resistant (MDR) infections worldwide. The lineage defined as sequence type (ST)-258 is a notorious example of MDR *K. pneumoniae*; ST‐258 frequently carries the *K. pneumoniae* carbapenemase (KPC) gene, as well as numerous other acquired AMR determinants [11].

*P. aeruginosa* is an opportunistic pathogen highly prevalent in hospital settings, particularly in patients under medical care. It thrives on biotic and abiotic surfaces, such as medical equipment, which is responsible for its biofilm-mediated drug resistance and the formation of multidrug-tolerant persistent cells associated with recalcitrance and relapse of infections [12]. It is mainly associated with hospital-acquired infections, ventilator-associated pneumonia, and central line-associated bloodstream infections [13]. *P. aeruginosa* is intrinsically resistant to various antimicrobials, such as β-lactams [14]. Drug efflux is a crucial resistance mechanism in Gram-negative bacteria such as *P. aeruginosa*. *P. aeruginosa* drug resistance is promoted by highly homologous three-component efflux systems of broad substrate specificity, four of which have been identified to date: MexA-Mexs-OprM, MexX-MexY-OprM, MexC-MexD-OprJ, and MexE-MexF-OprN [15]. This efflux system pumps antibiotics out of the bacterium and ensures that the intracellular antibiotic concentration does not reach minimum inhibitory concentration levels. Another drug resistance mechanism in *P. aeruginosa* is the production of β-lactamase, an enzyme that hydrolyses β-lactam antibiotics and leads to their inactivation.

Effect of COVID-19 on antimicrobial resistance

Some studies have shown that a minority of COVID-19 patients need antibiotics to treat secondary bacterial infections [15]. The lack of a standard therapy against the virus during the initial phase of the pandemic made many healthcare providers resort to the use of antibiotics to prevent and treat COVID-19 infections. However, these drugs were not proven to be scientifically effective in treating the virus but served as prophylaxis to the patients and seemed promising. This consequently led to the indiscriminate use of antibiotics, which further contributed to increased AMR in the general population. Current evidence reiterates the need to avoid antibiotic therapy or prophylaxis in patients with suspected or confirmed mild to moderate COVID-19 illness unless otherwise indicated [16].

Finally, MRSA's decreased susceptibility could be a pointer to the detriments of antibiotic misuse during the COVID-19 pandemic and warrants further collaborative multicenter studies [17].

Limitations

The main limitation of this study is that our dataset was limited since we only analyzed data spanning five years from a single center. The data were gathered and calculated from hospital antibiograms in whole numbers instead of rounded-up percentages.

## Conclusions

Based on our findings, certain organisms demonstrated a large drop in susceptibility to specific antibiotics, notably MRSA, while others showed a considerable improvement in susceptibility to antibiotics. The extent to which COVID-19 influenced these modifications is unknown. The observed alterations could be due to a variety of reasons other than COVID-19. Further research into antibiotic regulations and prescribing trends may shed more light on these issues. Other non-statistically significant findings in the study could be attributed to the limited data available to us.
